# Sarcoidosis: molecular mechanisms and therapeutic strategies

**DOI:** 10.1186/s43556-025-00244-z

**Published:** 2025-02-05

**Authors:** Danfeng Xu, Xiaohua Tao, Yibin Fan, Yan Teng

**Affiliations:** Center for Plastic & Reconstructive Surgery, Department of Dermatology, Zhejiang Provincial People’s Hospital, Affiliated People’s Hospital, Hangzhou Medical College, Hangzhou, Zhejiang China

**Keywords:** Sarcoidosis, Molecular mechanism, Therapy, Biological agents, Biomarkers

## Abstract

Sarcoidosis, a multisystemic granulomatous disease with unknown etiology, is characterized by formation of noncaseating granulomas, which can affect all organs. Recent studies have made outstanding achievement in understanding the pathology, etiology, genetics, and immune dysregulation involved in granuloma formation of sarcoidosis. Antigen stimulation in genetically predisposed individuals enhances the phagocytic activity of antigen-presenting cells, including macrophages and dendritic cells. CD4 + T cells initiate dysregulated immune responses and secrete significant quantities of inflammatory cytokines, including interleukin (IL)-2 and interferon-gamma (IFN-γ), which play a crucial role in modulating the aggregation and fusion of macrophages to form granulomas. The current therapeutic strategies focus on blocking the formation and spread of granulomas to protect organ function and alleviate symptoms. The efficacy of traditional treatments, such as glucocorticoids and immunosuppressants, has been confirmed in the management of sarcoidosis. Promising therapeutic agents encompass inhibitors of cytokines, like those targeting tumor necrosis factor (TNF)-α, as well as inhibitors of signaling pathways, such as Janus kinase (JAK) inhibitors, which exhibit favorable prospects for application. Although there has been progress in the identification of biomarkers for the diagnosis, prognosis, activity and severity of sarcoidosis, specific and sensitive biomarkers have yet to be identified. This review outlines recent advancements in the molecular mechanisms and therapeutic strategies for the sarcoidosis.

## Introduction

**Fig. 1:**
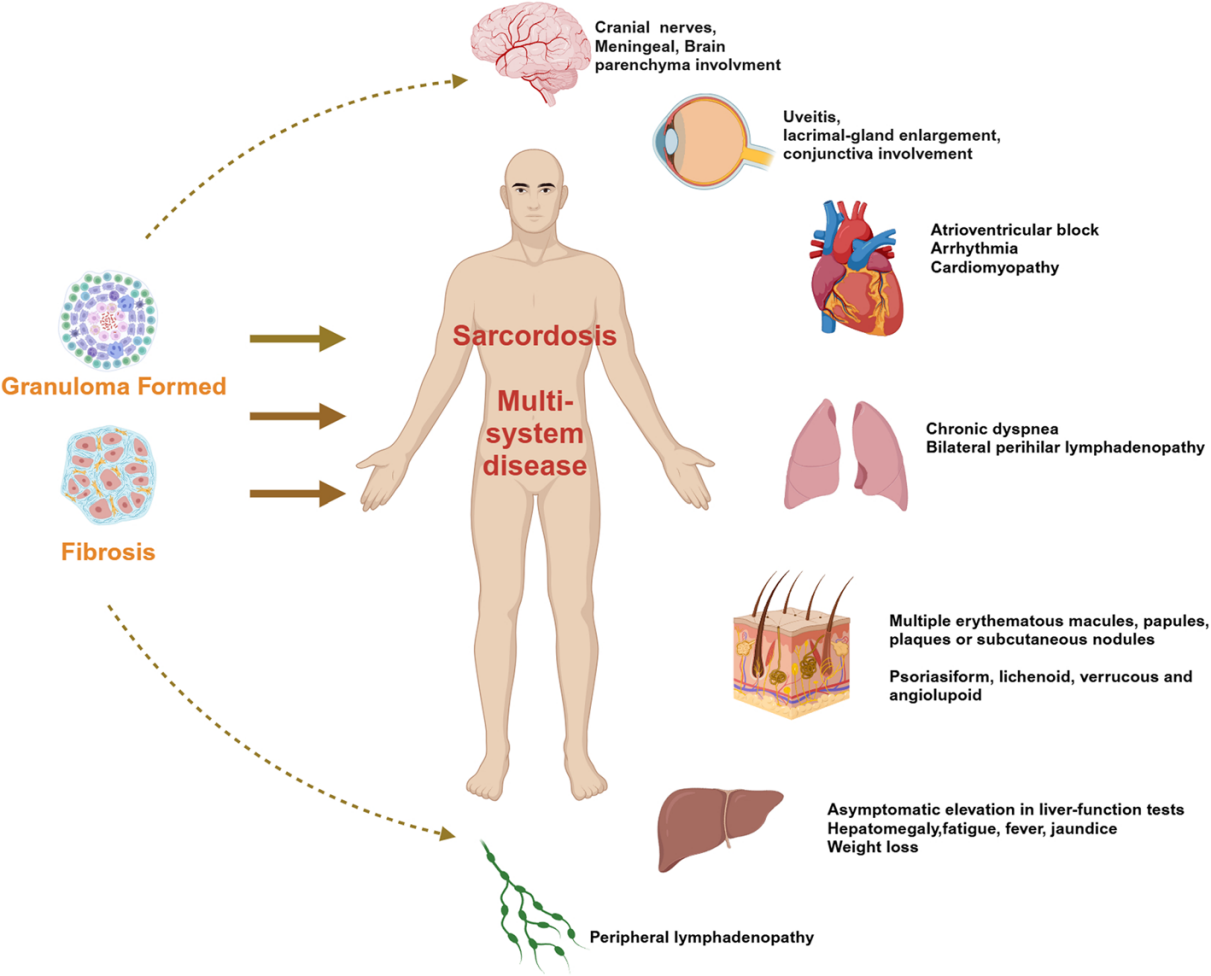
The systemic effects of sarcoidosis (Created with BioRender.com)

Sarcoidosis is an idiopathic systemic granulomatous disease characterized by the presence of confluent, non-necrotizing, and non-caseating granulomas. Sarcoidosis affects multiple systems and can involve various organs and tissues. The lungs are the most commonly affected site, followed by lymph nodes, skin, liver, eyes, heart, and even the central nervous system (Fig. 1) [1, 2]. Sarcoidosis exhibits an annual incidence that ranges from 1 to 15 cases per 100,000 people, depending on the geographical region. [3]. More than half of patients with sarcoidosis experience spontaneous remission following the acute phase. However, 10–30% of patients receive treatment, the therapeutic effect is often poor, leading to chronic inflammation and local fibrosis. Fewer than 10% will die from late-stage pulmonary or cardiac complications of sarcoidosis [4]. The unresolved chronic inflammatory response is the key pathogenic mechanism underlying sarcoidosis, inducing irreversible organ dysfunctions [5].

Sarcoidosis is influenced by a combination of various factors, including the individual’s genetic predisposition, environmental exposure to "unknown" antigens, and complex immune responses [6]. Currently, the diagnosis and treatment of sarcoidosis face significant challenges. On one hand, the complexity of its clinical manifestations and the unclear pathological molecular mechanisms contribute to a lack of reliable diagnostic biomarkers, leading to a high likelihood of misdiagnosis and missed diagnoses in the early stages of sarcoidosis [7]. On the other hand, oral glucocorticoids are utilized as the primary therapeutic option, while immunosuppressants are regarded as second-line therapies. However, some patients may experience adverse reactions associated with long-term use of these treatments. [8].

The main pathological mechanism of sarcoidosis involves antigen-presenting cells (such as macrophages and dendritic cells) that phagocytize antigens, leading to an abnormal immune response by CD4 + T cells. This results in the secretion of inflammatory cytokines like IL-2 and IFN-γ, causing macrophage aggregation and fusion into granulomas [9]. Recent studies have found that the pathological mechanisms of sarcoidosis also involve other pro-inflammatory factors (such as TNF-α) and multiple signaling pathways (such as mTOR and JAK-STAT pathway). These findings suggest new promising therapeutic targets for sarcoidosis [10, 11]. Recent advancements in the research of autoimmune molecular mechanisms and novel therapeutic strategies for sarcoidosis have provided new insights for the comprehensive management of sarcoidosis [12].

In this review, we offer a novel and comprehensive perspective on recent advancements in the molecular mechanisms of sarcoidosis and its therapeutic implications [13, 14]. We aim for this review to further investigate promising therapeutic targets and potential biomarkers that may be employed for diagnosing or predicting disease progression and prognosis.

## Clinical features

Most early cases of sarcoidosis are asymptomatic or present with nonspecific symptoms, making misdiagnosis or missed diagnosis common. Dry cough, shortness of breath, and chest discomfort are the most common clinical symptoms, occurring in about 30–50% of patients and more prominently in pulmonary sarcoidosis where lesions invade lung tissue or manifest as bronchial involvement [15]. Systemic symptoms such as fatigue, weight loss, and night sweats are also quite common. Most patients with pulmonary sarcoidosis are identified during a routine chest radiograph, with up to 50% of cases detected in this manner. The severity of pulmonary sarcoidosis is then classified based on imaging findings, including lung infiltration and/or lymphadenopathies [16]. Table 1 provides a detailed description of the Scadding stage criteria used to assess the severity of sarcoidosis. Löfgren syndrome represents a distinct phenotype characterized by acute symptoms including fever and erythema nodosum, which may be accompanied by arthritis. This type is often associated with a favorable prognosis [17].

**Table 1:** Standards for the scadding stage of sarcoidosis

Radiographic Type	Radiographic Characteristics
0	No visible findings
I	Bilateral hilar lymphadenopathy
II	Bilateral hilar lymphadenopathy and parenchymal infiltration
III	Parenchymal infiltration without hilar adenopathy on chest X-ray
IV	Advanced fibrosis with significant distortion of normal lung architecture, primarily affecting the middle and upper lobes. This condition includes bronchiectasis, hilar retraction, bullae, cysts, and less commonly "honeycombing."

Due to sarcoidosis being a multi-system disease, patients with different organ involvement may exhibit specific symptoms. According to ACCESS data, 95% of patients have pulmonary manifestations, 50% have extrapulmonary symptoms, and 2% present with isolated extra-thoracic sarcoidosis [18]. Therefore, for sarcoidosis, further research on molecular biomarkers for diagnosis and prognostic prediction, as well as treatment strategies, needs to be linked with the types of affected organs and the severity of the disease.

## Pathology of sarcoidosis

Granulomatous infiltration and fibrosis of organs or tissues in sarcoidosis can be progressive and life-threatening [19]. The occurrence of respiratory failure alongside cardiac, neurological, renal, and progressively fibrotic pulmonary involvement is linked to heightened morbidity and mortality rates.

The classical histopathological feature of sarcoidosis is chronic granulomatous inflammation. Granuloma is non-necrotizing with a tightly packed central region comprising multinucleated giant cells, epithelioid cells, macrophages, and CD4 + T lymphocytes [20]. The central region is surrounded by CD4 + and CD8 + T lymphocytes, B lymphocytes, mast cells, fibroblasts, and monocytes. The common characteristic of sarcoid granuloma is the development of ring fibrosis, which is subsequently replaced by hyaline and dense scars [21]. Scar tissue can persist for a prolonged duration, while persistent fibrosis is observed in some typical organs, such as lungs and the skin. The proportion of lymphocyte infiltration and fibrosis around granuloma varies depending on the patient and disease duration [22, 23]. Other histopathological features of sarcoidosis granulomas include the presence of asteroid bodies, Schaumann bodies, as well as birefringent crystalline structures [24].

The other histopathological types of sarcoidosis are necrotizing sarcoid granulomatosis and nodular sarcoidosis [25]. Necrotizing sarcoid granulomatosis, which mainly affects the lungs and also is rare, is characterized by perivascular masses comprising confluent granulomas and lung parenchyma necrosis [26]. Nodular sarcoidosis is characterized by confluent non-necrotizing granulomas in large masses, always surrounded by extensive fibrosis [27].

## Molecular mechanisms of sarcoidosis

Given the unknown etiology of this granulomatous disease, sarcoidosis cannot be categorized as an autoimmune disorder. However, certain studies have indicated that immune disorders significantly contribute to the development of sarcoidosis. [28, 29]. Specific auto-antigens or auto-antibodies have not yet been identified in sarcoidosis. Sarcoidosis exhibits characteristics resembling those of autoimmune diseases, notably its association with human leukocyte antigen (HLA) genotypes. [30], favorable responses to immunosuppressive therapy, and frequent co-occurrence with autoimmune diseases [31–35]. The complex immunological features of sarcoidosis are related to genetic factors and environmental exposure [36]. The elucidation of the autoimmunity mechanism in sarcoidosis can enable the application of targeted immunotherapy and aid in the identification of key biomarkers in patients with sarcoidosis [37, 38].

### Genetic predisposition to sarcoidosis

The occurrence of sarcoidosis in families was first confirmed in a multicenter study in US involving 291 African Americans, 355 European Americans, and 27,000 first-degree and second-degree relatives [39]. Recent epidemiological studies revealed that the incidence and clinical manifestations of sarcoidosis vary among ethnic groups [40, 41], suggesting genetic susceptibility to sarcoidosis.

Genetic predisposition towards sarcoidosis is mostly linked to the HLA-encoding genes within the major histocompatibility complex (MHC) situated on chromosome 6 [42]. Both HLA class I and HLA class II are associated with an increased risk of sarcoidosis. The HLA region is linked to over 100 complex diseases, primarily related to autoimmune and inflammatory processes. The granulomatous response in sarcoidosis is triggered by the three-component complex of T-cell receptor (TCR)-peptide-HLA, serving as the initial stimulatory signal for antigen-specific T cells, followed by stimulation from co-stimulatory molecules as a second signal. [43].

HLA class I and II molecules present a wide range of antigenic peptides to T cells, thereby facilitating the immune system’s ability to distinguish between self and foreign antigens. During autoimmune diseases, autoreactive T cells circumvent negative selection within the thymus and escape the peripheral tolerance mechanisms tied to disease-specific HLA-peptide-TCR complexes, ultimately resulting in tissue injury. Recent studies have revealed new mechanisms, such as low-affinity peptide binding aiding thymic evasion, unusual binding orientations of HLA-peptide-TCR, T cell receptor-mediated stabilization of weak peptide-HLA complexes, presentation of peptides in diverse binding arrangements, "hotspot" molecular mimicry, and post-translational modifications [44]. These offer additional research avenues for exploring the HLA gene’s molecular mechanisms in sarcoidosis.

The mycobacterial catalase-peroxidase protein (mKatG) has been detected in a majority of sarcoidosis samples and is reported to preferentially elicit a response from TCR AV2S3 + T cells in the lungs of HLA-DRB1*03 + patients. [45, 46]. Wahlström et al. isolated peptides from bronchoalveolar lavage (BAL) cells sourced from 16 sarcoidosis patients carrying the HLA-DRB1*0301 allele. Among these peptides were those originating from established autoantigens, notably ATP synthase and vimentin [47]. Although the molecular mechanisms linking HLA genes to the pathogenesis of sarcoidosis remain unclear, some studies suggest that autoimmunity associated with HLA genes may contribute to the inflammation observed in sarcoidosis. [43, 48].

Several HLA alleles have been identified with disease phenotypes in sarcoidosis [49].HLA-DRB1*01 and HLA-DRB1*04 exhibit a negative correlation with sarcoidosis, suggesting they confer a protective role against the disease. In contrast, HLA-DRB1*03, HLA-DRB1*11, HLA-DRB1*12, HLA-DRB1*14, and HLA-DRB1*15 are linked to an increased susceptibility to sarcoidosis [50]. Certain HLA alleles are related to the progression of sarcoidosis. For example, HLA-DRB1*0301 is associated with the abrupt onset of sarcoidosis and a rapid, spontaneous resolution of the disease [51]. Previous studies revealed that HLA-DRB1*1501 and HLA-DRB1*0602 are correlated with a chronic course, while HLA-DRB1*150101 and HLA-DRB1*0602 are related to severe pulmonary sarcoidosis [52, 53].

It has been identified in several Genome-wide linkage analyses that some additional genes are associated with increased susceptibility to sarcoidosis. A variation in *BTNL2,* situated in proximity to the HLA-encoding genes on chromosome 6, was found to be associated with sarcoidosis. This variation, a single nucleotide polymorphism (rs2076530 G → A) in *BTNL2* leads to the production of a truncated variant and is associated with the CD80 and CD86 co-stimulatory receptors, which play a crucial role in regulating T lymphocyte activity [54, 55]. Genome-wide association study in sarcoidosis was first performed in Germany by Hofmann et al. who illustrated the connection between *ANXA11* rs1049550 and sarcoidosis [56].Recent studies revealed that angiotensin-converting enzyme (ACE) variants are associated with the occurrence and severity of sarcoidosis [57].

However, the genetic susceptibility of sarcoidosis is complex and appears to be race-specific. Recent expression quantitative trait loci (eQTL) and genome-wide association studies (GWAS) investigations highlight the significance of MHC Class I genes, specifically HLA-B and HLA-C, in European Americans. In contrast, African Americans primarily exhibit associations with the HLA-B gene [58, 59].

Previous research has established the role of genetic elements in the pathogenesis of sarcoidosis. Various gene loci are reported to be correlated with the susceptibility, progression, and prognosis of sarcoidosis. However, genetic loci that can accurately diagnose sarcoidosis with sufficient sensitivity and specificity have not been identified [5]. Further studies are needed to discover the genetic characteristics of sarcoidosis and consequently develop novel molecular biomarkers for sarcoidosis.

### Etiology of sarcoidosis

**Fig. 2:**
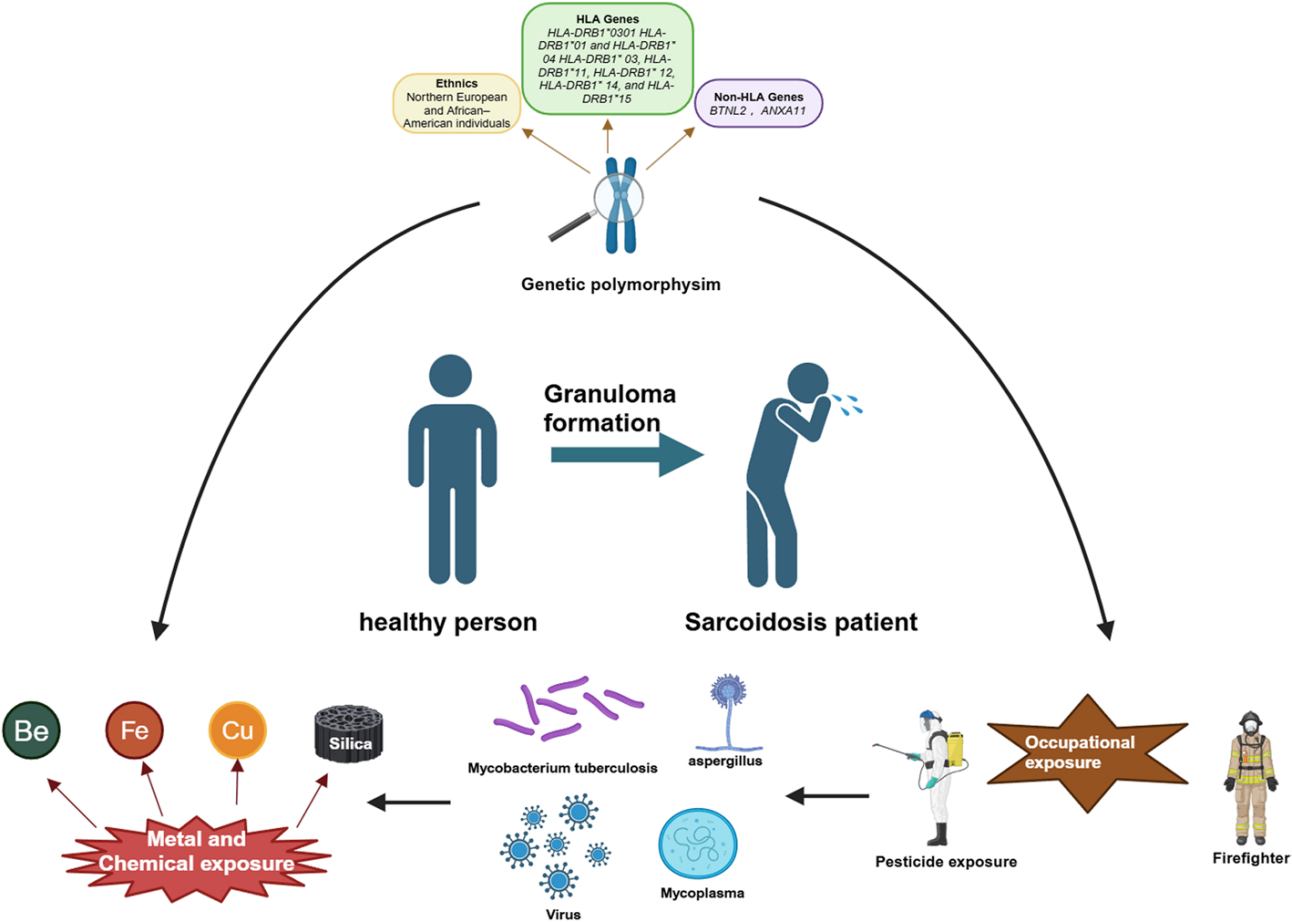
Granuloma formation in sarcoidosis is influenced by a range of etiological factors; The formation of granulomas in sarcoidosis is influenced by genetic susceptibility factors related to race, HLA, and non-HLA genes, as well as infectious pathogen and environmental or occupational exposures. Some patients may experience spontaneous remission within a year, while others may progress to a chronic course (Created with BioRender.com)

Granulomatous inflammation in sarcoidosis is caused by excessive cell-mediated immune responses against some unidentified antigens [60]. Granuloma formation in sarcoidosis is multifactorial, involving genetics, infections, and environment. Disease course varies, with some patients experiencing spontaneous remission and others developing chronicity (Fig. 2). Recent studies have identified specific etiological agents discussed in this section.

#### Environmental and occupational exposure

Several epidemiological studies have demonstrated that one of the etiological factors for sarcoidosis is exposure to organic bioaerosols, such as musty odors in the workplace and industrial organic dust [61, 62]. It was observed that siblings diagnosed with sarcoidosis were more likely to report indoor exposure to musty odors compared to their unaffected family members in a case–control etiologic study of sarcoidosis survey (ACCESS) [62]. Additionally, exposure to silica and inorganic aerosols, especially metal dust, such as beryllium, copper, and iron is reported to be correlated with sarcoidosis [63–65]. Sarcoidosis was frequently observed in firefighters but not in emergency medical technicians (who were used as a control group in fire sites) [66], suggesting that exposure to combustible products is a potential etiological factor for sarcoidosis. It is consistent with previous findings, which reported that the incidence of sarcoidosis due to dust exposure during the 4 years following the World Trade Center disaster was higher than that during the 4 years preceding the disaster [67]. A meta-analysis found that occupational silica exposure was linked to pulmonary sarcoidosis (OR 1.26, 95% CI 1.02–1.56, I^2^ 33.7%). Correspondingly, mould or mildew exposure (OR 1.52, 95% CI 1.21–1.91, I^2^ 0%) and occupational pesticide exposure (OR 1.42, 95% CI 1.09–1.85, I^2^ 14.3%) were also linked to the diagnosis. Organic dust was reported in a single case–control study only (OR 2.57, 95% CI 1.35–5.16). [68].

The prevalence of sarcoidosis is high in northern Europe and northern Japan, which can be attributed to decreased sunlight exposure, resulting in 1, 25-dihydroxy-vitamin D deficiency. And the deficiency of 1,25-dihydroxy-vitamin D leads to a decrease in the production of the antimicrobial peptide, which promotes the development of infectious granulomatous disease [69, 70].

Thus, sarcoidosis is associated with various environmental and occupational exposures, which activate the specific immune pathways of sarcoidosis by stimulating the immune system through different mechanisms. Genetic factors may play a role in this mechanism [71]. Recent research indicates that sarcoidosis stems from an intricate interaction of genetic, immune, and environmental factors. For instance, the link between HLA genotypes and occupational exposures is associated with specific sarcoidosis phenotypes, such as extrapulmonary disease. [72, 73]. However, to date, there have been few studies that link genetic research with environmental exposures, indicating a need for further investigation in this area.

#### Pathogen infection

Infections from several pathogens, especially *Mycobacterium* and *Propionibacterium acnes*, are suspected to be associated with sarcoidosis [74, 75]. A meta-analysis of infection-related sarcoidosis suggested that mycobacteria are potential etiological agents for sarcoidosis [76]. Molecular analysis revealed the presence of mycobacterial genes in granulomas [74, 77]. Various tuberculosis-associated antigens can stimulate peripheral blood mononuclear cells (PBMCs) of sarcoidosis patients, eliciting type 1 T helper cell (Th1) immune responses. In phase II, multicenter, double-blind, randomized controlled clinical trial involving 97 patients with pulmonary sarcoidosis, concomitant treatment with ethambutol, levofloxacin, azithromycin, and rifabutin for 16 weeks did not significantly improve pulmonary function index [78]. Many previous studies suggest that *Mycobacterium tuberculosis* infection is associated with granulomatosis in patients with sarcoidosis [79, 80]. However, the presence of *M. tuberculosis* in the lesions has not been confirmed using isolate culturing or tissue staining. Additionally, anti-tuberculosis therapy did not exert beneficial effects in patients with sarcoidosis. Thus, *Mycobacterium*-induced immune response may be the etiological factor for sarcoidosis rather than mycobacterial infection [81, 82].

*Propionibacterium acnes* is also associated with the pathogenesis of sarcoidosis as evidenced by the presence of pathogen genetic and protein materials in the lesions [83–85]. However, antimicrobial agents do not exert therapeutic effects on sarcoidosis [86]. The pathogenesis of sarcoidosis is reported to involve the intracellular proliferation of reactivated *P. acnes* at the latent infection sites in predisposed individuals with Th1 hypersensitivity, which is potentially mediated by the *NOD1* allele type. Extracellular *P. acnes* after intracellular proliferation promotes new latent infection in various organs, contributing to the formation of granuloma [87, 88]. Therefore, *P. acnes* can promote sarcoidosis through endogenous hypersensitivity infections by activation of Th1 immune responses.

Microbes could potentially have a significant impact on the development of sarcoidosis through their regulation of metabolic and immune processes [85]. The pathogenesis of sarcoidosis caused by pathogen infection is unclear. The elucidation of the mechanism of these external and internal factors involved in the pathogenesis of sarcoidosis will aid in revealing the molecular pathological mechanism of sarcoidosis.

### Mechanism of granulomatous inflammation

**Fig. 3:**
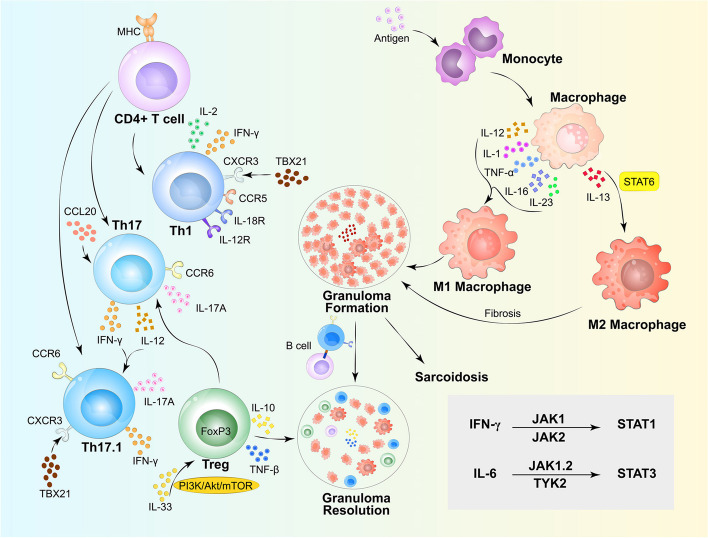
Molecular mechanisms of granulomatous inflammation for sarcoidosis; Antigen-presenting cells, like macrophages and dendritic cells, initiate phagocytosis of antigens. Dysregulation of autophagy and phagocytic lysosomal pathways alters macrophage function, leading to aggregation and granuloma formation. Inflammatory cytokines such as IFN-γ, IL-2 are linked to the increase of effector TH1 cells that activate classically activated macrophages(M1). Interleukin-17-producing T helper (Th17) cells can differentiate into IFN-γ-producing Th17 (Th17.1) cells when stimulated by IL-12 and IFN-γ. Th17.1 cells express CC chemokine receptor 6 (CCR6) (Th17 receptor) and CXC chemokine receptor (CXCR) 3 (Th1 receptor), secreting IL-17A or IFN-γ. Treg cells modulate inflammation through direct interactions or by secreting cytokines like IL-10 and transforming growth factor β (TGF-β). IL-33 can modulate Treg function via the mammalian target of rapamycin (mTOR)1 signaling pathway, and cytokines such as IFN-γ regulate the JAK signaling pathway, thereby influencing granuloma formation

The response of granulomatous inflammation is a key process in the pathogenesis of sarcoidosis. Pathogen infection and autoantigen or inorganic antigen stimulation in genetically susceptible individuals promote the phagocytosis of antigens by antigen-presenting cells, such as macrophages and dendritic cells. CD4 + T cells elicit aberrant immune responses and secrete large amounts of inflammatory cytokines, such as IL-2 and IFN-γ. The dysregulation of the autophagy and phagocytic lysosomal pathways changes the functional state of macrophages, which aggregate and fuse to form granuloma [89, 90]. The detailed molecular mechanisms underlying the formation of granulomas in sarcoidosis are illustrated in Fig. 3. Additionally, Sarcoidosis, a granulomatous disorder, is distinguished by the concentrated and targeted migration of granuloma cells, including lymphocytes. Therefore, any changes in their relative abundance in peripheral blood can be attributed not only to alterations in their production but also to selective redistribution following their migration to granulomatous lesions [91].

#### Macrophages and dendritic cells

Macrophages play an essential role in all aspects of sarcoidosis granuloma formation [92]. Macrophages serve a dual role: they initiate and amplify adaptive immune responses, while also participating in antigen presentation, pro-inflammatory reactions, and constituting a fundamental element in granuloma formation. Upon stimulation by bacterial, inorganic, or self-antigens, macrophages initiate the adaptive immune response and lead to the substantial production of serum amyloid A (SAA), thereby inducing acute inflammation [93]. This inflammatory response fosters an environment conducive to sustained inflammation and encourages immune cell involvement in granuloma formation.

Based on their functional characteristics, macrophages can be broadly classified into two types: classically activated macrophages (M1) and alternatively activated macrophages (M2). The polarization of M1 macrophages is generally triggered by the synergistic action of inflammatory cytokines, such as IFN-γ and TNF, along with Toll-like receptors (TLRs) [94]. M1 macrophages play a major role in clearing antigens and mediating innate and adaptive immune responses and other defense processes. M2 macrophages inhibit excessive inflammatory responses and maintain immune homeostasis by releasing inflammatory factors, such as IL-10 [95, 96]. In the early stage of sarcoidosis, M1 macrophages and monocytes are activated upon antigen stimulation. The antigens are endocytosed and presented to T cells via MHC. Several cytokines, such as IL-12, IL-1, IL-16, IL-23 and TNF-α promote the recruitment of immune cells into the inflammation site. TNF-α plays an important role in the initial T cells (Th0) polarization toward the Th1 phenotype. Subsequently, monoclonal proliferation and secretion of IFN-γ promote M1 activation. The ineffective clearance of antigens promotes macrophage proliferation, leading to the formation of granuloma. IFN-γ plays an important role in maintaining M1 macrophage activity. Thus, sarcoidosis has been considered a Th1/M1-mediated disease [97, 98].

Recent studies have reported that M2 macrophages may mediate the formation of granuloma in sarcoidosis. Analysis of sarcoidosis-affected neuromuscular tissues revealed that M2 macrophages are activated by alternative pathways based on surface markers. Additionally, M2 macrophage activation was associated with fibrosis and C–C motif chemokine ligand (CCL) 18 upregulation. Immunohistochemical comparison of lymph node tissues from patients with tuberculosis and sarcoidosis indicated a notable upregulation of CD163 expression in sarcoidosis cases, implying the involvement of M2 macrophages in the development of sarcoidosis granulomas [99, 100]. A recent study suggested that IL-13 is the most important cytokine regulating differential expression in sarcoidosis and that M2 polarization is mediated via IL-13-induced activation of signal transducers and activators of transcription (STAT)6. Pretreatment of PBMCs with STAT-6 inhibitors significantly decreased granulomatous nodules and M2 polarization, which provided a novel strategy to clarify the role of M2 macrophages in sarcoidosis. Blocking the chronic fibrosis process by inhibiting M2 polarization is a potential novel therapeutic strategy for sarcoidosis [101–103].

Chen et al. discovered an upregulation of TLR2 expression in the mediastinal lymph nodes of patients with sarcoidosis. [104]. TLR2 triggers the activation of nuclear factor kappa-B (NF-κB) via IL-1R-associated kinase 4 (IRAK4), IL-1R-associated kinase 1 (IRAK1), and tumor necrosis factor receptor-associated factor 6 (TRAF6), ultimately leading to the expression of inflammatory mediators and chemokines, namely chemokines chemokine (C-X-C motif) ligand(CXCL) 8 and CCL20 [105]. This indicates the potential of TLR2 as a marker for predicting sarcoidosis activity. Additionally, Abedin et al. revealed a significant increase in serum levels of IL-4 and IL-13 cytokines in sarcoidosis patients versus healthy individuals [106]. IL-4 and IL-13 have the potential to enhance macrophage fusion and augment their phagocytic capabilities via the STAT6 signaling pathway [107]. Talreja et al. found macrophage migration inhibitory factor (MIF) induce Mitogen-activated protein kinase phosphatase (MKP)-1, Tregs, IL-10 and IL-1RA to modulate mitogen-activated protein kinases (MAPK) activation [108].

Dendritic cells (DCs) are also implicated in the aberrant immune mechanisms leading to granuloma formation in sarcoidosis. Experimental evidence has established the crucial role of DCs as antigen-presenting cells in triggering the subsequent adaptive immune response in sarcoidosis patients [109]. Recent investigations of tissue samples from sarcoidosis patients have demonstrated that mature dendritic cells (DCs) are primarily aggregated in draining lymph nodes, and they exhibit a stronger capacity to stimulate T-cell clonal proliferation compared to alveolar macrophages. [110]. Lepzien et al. revealed that DCs from sarcoidosis patients into Th1 and Th17.1 subsets, leading to the production of IFN-γ, which is implicated in disease progression [111]. Therapies targeting DCs exhibit promising potential for future applications, however, additional research is necessary to decipher the molecular mechanisms that govern the interaction between DCs and sarcoidosis.

#### Th1/Th2 cells

Histopathologically, CD4 + T cells are situated in the outer layer of granulomas and exert a pivotal role in sarcoidosis [112].Upon recognizing and being activated by antigens, CD4 T cells possess the capacity to differentiate into diverse subsets, encompassing Th1, Th2, Th17, Th17 cells producing IFN-γ, as well as various Tregs, which can either suppress or enhance granulomatous inflammation [113]. Contemporary research indicates that sarcoidosis constitutes a dynamic process implicating the regulation of the Th1/Th17/Th2 balance as well as dysfunction of Treg/Th17 types in the formation of sarcoidosis granulomas [114, 115].

Th1 cells were considered to be the main effector T cells in early research. In patients of active pulmonary sarcoidosis, IL-2 and IFN-γ are spontaneously released by lung lymphocytes. T lymphocytes present in the lung and blood spontaneously express functional IL-2 receptors (IL-2R) [116, 117]. This indicates that Th1 immune response is correlated with the formation of sarcoid granuloma because both IL-2 and IFN-γ are vital Th1 cytokines [118]. Recent studies examining cytokine and chemokine expression in sarcoidosis have confirmed the association between Th1 immune response and sarcoidosis. In the BAL fluid of patients with sarcoidosis, an upregulation is observed in inflammatory cytokines, specifically TNF-α, IFN-γ, IL-12, and Macrophage Inflammatory Protein-1α (MIP-1α), alongside the chemokines CXCL9 and CXCL10.These cytokines and chemokines are closely linked to the elevation of effector Th1 cells within the lungs affected by inflammation [119, 120]. These findings suggest a pro-inflammatory condition and a preference towards the Th1 subtype of CD4 T-cells in sarcoidosis patients, with Th1 cells classically activating M1 macrophages. TNF-α is a key Th1 cytokine that acts synergistically to provide protective immunity to patients through mechanisms such as macrophage activation, granuloma formation, and maintenance [121]. In multiple randomized controlled trials, targeted therapy directed against TNF-α for sarcoidosis treatment has shown promising application potential [10]. In sarcoidosis patients, IFN-γ may exert anti-apoptotic effects on macrophages by inducing the expression of the cyclin-dependent kinase inhibitor p21/Waf1, thereby contributing to the persistence of lung inflammation [122].

Compared to peripheral blood, T cells accumulated in the lungs of sarcoidosis patients exhibit upregulated expression levels of CXCR3, CCR5, IL-18R, and IL-12R, suggesting the translocation of Th1 cells from the bloodstream into the lungs [123]. The bronchoalveolar lavage cell levels of *TBX21* mRNA in patients with sarcoidosis are higher than those in controls. TBX21, a Th1 cell transcription factor, can upregulate genes encoding IFN-γ and CXCR3 involved in sarcoidosis [124]. Therefore, further investigation into Th1-associated chemokines, cytokines, their respective receptors, and transcription factors may not only aid in elucidating the immunopathogenesis of sarcoid granulomas but also facilitate the identification of potential biomarkers indicative of sarcoidosis.

Moreover, patients recovering from sarcoidosis regain normal functional Th1 cell responses, whereas those with ongoing disease demonstrate irregularities in their intracellular signaling pathways. Oswald-Richter et al. found that peripheral CD4 T-cells from sarcoidosis patients secrete more IL-2 and IFN-γ compared to healthy controls. Upon polyclonal TCR stimulation, a notable decrease is observed in the secretion of IL-2 and IFN-γ, as well as in the expression of crucial transcriptional mediators of IL-2, such as lymphocyte-specific tyrosine kinase (Lck), protein kinase C-theta (PKC-θ), and NF-κB [125]. Braun et al. discovered that CD4 T-cells from sarcoidosis patients, which express Programmed Death-1 (PD-1), exhibited reduced proliferative capacity upon PD-1 blockade by antibodies. PD-1 negatively modulates proliferation, cytokine secretion, and survival of CD4 T-cells. Furthermore, patients who underwent spontaneous disease remission demonstrated a notable decrease in PD-1 expression, contrasting with those who had active disease [126]. The aforementioned observations suggest that the Th1 cell response is a pivotal factor in the development of sarcoidosis.

Furthermore, TH2-type immune responses have been associated with less favorable outcomes in sarcoidosis patients. Elevated levels of Thymus-and-Activation-Regulated Chemokine (TARC) and an increased percentage of cells expressing Chemokine Receptor type 4 (CCR4) were observed in patients with more extensive organ involvement and advanced stages of sarcoidosis, relative to those with milder disease manifestations. TARC is one of the CC chemokines, activates CCR4 which is selectively expressed on Th2 cells [127]. TH2 cells release cytokines like IL-4 and IL-13, which stimulate M2 macrophages, thereby facilitating the activation and proliferation of fibroblasts [128]. This could provide insight into the mechanistic role of TH2 in sarcoidosis.

Based on the aforementioned research, Th1/Th2 biomarkers hold potential value in predicting the diagnosis, prognosis and severity of sarcoidosis. However, the precise specificity and sensitivity of these biomarkers for sarcoidosis remain to be established, necessitating further studies to elucidate this matter.

#### Th17 cells

Contemporary research has indicated that Th17 cells play a role in the pathogenesis of sarcoidosis. CD4 T cells, specifically the TH17 subtype, are crucial in recruiting neutrophils to fight bacterial and fungal infections, thus enhancing the inflammatory response. Additionally, these cells may also cause autoimmunity [129]. These mechanisms could elucidate their participation in the formation of sarcoidosis granulomas.

One study reported that the number of IL-17A + /CD4 + T cells in the blood, bronchoalveolar lavage fluid (BALF), and sarcoid tissue and the expression of IL-17A in the sarcoid tissue are upregulated [130]. Additionally, the results of the migration assays demonstrated that Th17 cells can respond to the chemotactic stimulus CCL20 [131]. Sarcoidosis is currently considered to be a Th1 /Th17-mediated disease. The upregulation of IL-17A may be associated with a favorable prognosis for Löfgren syndrome [132].

Th17 cells can be further differentiated into Th17.1 cells upon stimulation with IL-12 and IFN-γ. Th17.1 cells express CCR6 (Th17 cell chemokine receptor) and CXCR3 (Th1 cell chemokine receptor) and secrete IL-17A and IFN-γ or only IFN-γ [133]. These cells stably express both RORγt (a key transcription factor of Th17 cells) and TBX21 at the single cell level, showing that Th17.1 cells have the proinflammatory ability of both Th1 and Th17 cells [134, 135]. Recent studies demonstrated that Th17.1 cells are the main producers of IFN-γ rather than Th1 cells in the BALF samples of patients with sarcoidosis [136]. Notably, although a higher proportion of TH17.1 CD4 T-cells was observed in the BAL fluid and mesenteric lymph nodes (MLN) of sarcoidosis patients, this was not mirrored in their peripheral blood. Instead, an elevated frequency of TH17 cells was detected in the peripheral blood of these patients. Upon migrating into inflamed lung tissue and encountering elevated levels of IL-12 and/or IFN-γ, circulating TH17 cells are inferred to differentiate into TH17.1 cells [136, 137]. Additionally, sarcoid T-cells in the peripheral blood, characterized as CCR4 + , CXCR3 + , and CCR6 + , which signify an intermediary phase between classical TH17 and TH17.1 cells, displayed reduced cytotoxic T lymphocyte-associated protein (CTLA)-4 expression levels compared to normal. In sarcoidosis patients, the lack of CTLA-4 in these cells may promote their increased proliferation within the lungs. When compared to patients experiencing disease resolution, an increase in the TH17.1 subtype and an elevated ratio of TH17.1 to TH17 cells were detected in the BAL fluid of individuals in the chronic course of sarcoidosis. [138]. Interestingly, the ratio of Th17.1 cells was inversely correlated to pulmonary function during follow-up [139]. Furthermore, multiple studies indicate that impaired TH17 cells might be implicated in the development of fibrotic pulmonary sarcoidosis. Elevated levels of PD-1, STAT3, and/or TGF-β1, along with a preponderance of TH17 infiltrates in the lungs, may serve as indicators of pulmonary fibrosis progression in sarcoidosis patients [140]. Therefore, although the mere presence of TH17 or TH17.1 cells is not sufficient as a conclusive marker for sarcoidosis, changes in TH17 and TH17.1 cell levels, combined with increased amounts of TGF-β1, IFN-γ, or IL-17A, can provide valuable insights into disease severity and progression.

#### Treg cells

Treg cells, a subset of CD4 T-cells that respond to both self and foreign antigens, play a crucial role in the immune system. They possess significant immunosuppressive capabilities towards Th cells, B cells, and various other immune cell types [141]. Treg cells modulate inflammation through mechanisms involving direct cell–cell interactions or the secretion of cytokines, such as IL-10 and TGF-β. Furthermore, their differentiation is dependent on the transcription factor Forkhead box P3 (FoxP3) [142]. Tregs were observed to be elevated in the peripheral blood of patients with sarcoidosis, while their levels remained within normal ranges in BAL fluid from individuals with the condition [143, 144]. However, the reasons for the elevated levels of Treg cells in sarcoidosis, particularly in chronic sarcoidosis, remain unclear.

Treg cells can inhibit the proliferation of CD8 + and CD4 + cells by secreting immunosuppressive factors, such as TGF-β and IL-10 through FoxP3 or by directly interacting with CD25 on the membrane surface to suppress the degree of immune response [145]. It is reported that the proportion of circulating Treg/Th17 cells is negatively correlated to sarcoidosis activity and that the ratio of Treg/Th17 cells are upregulated in patients who achieve remission but return to physiological levels after treatment [146]. Acknowledged as the chief mechanism enabling Treg cells to inhibit other lymphocytes, CTLA-4 functions as a pivotal indicator of Treg cell functionality. The decreased CTLA-4 expression observed in MLN Treg cells implies a potential impairment of Treg cell function upon their migration into the chronic inflammatory milieu of the lungs. The concurrent absence of CTLA-4 on both TH17 and Treg cells may potentiate TH17 or TH17.1 cell activation, while concurrently hindering the suppressive functions of Treg cells. This phenomenon may clarify increased proportion of TH17/Treg cells observed in sarcoidosis, as well as the rebalancing of Th17/Treg cells following treatment [141, 147]. Furthermore, some studies have reported that the upregulation of circulatory levels of Treg cells at the early stage of diagnosis may be related to chronic sarcoidosis. Additionally, the increased levels of Treg cells increase the risk of autophagy and dysfunction [144, 148].

Zhang et al. [149] demonstrated that IL-33 can alleviate Treg cell dysfunction in the sarcoidosis mouse model through the upregulation of IL1RL1 and the inhibition of PI3K/Akt/mTOR and TGF-β/Smad2/Smad3 signal pathways. Dysregulation of the PI3K/Akt/mTOR signaling pathway was observed in lung tissue and T cells upon administration of a PI3K/Akt/mTOR pathway inhibitor. And the immunosuppressive function of Treg cells was restored after blocking this pathway, suggesting that inhibiting the P13K/Ak signaling pathway can alleviate Treg cell dysfunction in sarcoidosis [150]. Zhang et al. revealed that administering CAL-101 (PI3Kδ-specific inhibitor) and AS-605240 (dual PI3Kδ/γ inhibitor) via transtracheal route promoted pulmonary granuloma formation by inhibiting PI3Kδ/γ, consistent with earlier observations. [151].

Enhancing the treatment of sarcoidosis through the modulation of Th17/Treg rebalancing presents a promising avenue for future research. Additionally, this approach may act as a potential biomarker for monitoring disease progression and prognosis.

#### B cells

In recent years, the involvement of B cells in the formation of granulomas associated with sarcoidosis has been recognized to have a greater impact than previously understood [152]. Nevertheless, the involvement of B cells in the pathophysiology of sarcoidosis remains elusive.

An increase in the number of B lymphocytes and their derivative plasma cells is noted in the vicinity of granulomas. [153]. There are less circulating memory B cells, more transitional and IL-10-producing regulatory B cells in active chronic sarcoidosis patients [154]. And case reports of rituximab decreasing inflammation in sarcoidosis patients also suggest that B cells have a certain effect in the pathogenesis of sarcoidosis [155, 156]. T follicular helper (Tfh) cells have high levels of the chemokine receptor CXCR5 on their membrane, which is essential for their migration to B cell follicles rich in CXCL13 [91]. Reports indicate that Tfh2 and Tfh17 CD4 T cells stimulate B cells to secrete IgM, IgG, and IgA via IL-21, whereas Tfh1 cells induce apoptosis in activated B cells [157]. Regulatory B cells (Bregs), characterized by CD5 CD27 +− and CD24 CD38 markers and IL-10 production, augment the levels of suppressive cytokine IL-10. This elevation in IL-10 can downregulate TH1 responses, thereby promoting non-inflammatory and fibrotic repair processes [91].

B cell-Activating Factor (BAFF), as a member of the TNF-ligand family and associated in the development of B cells, is found significantly increases in peripheral blood of sarcoidosis patients. Another study showed that serum BAFF levels were positively correlated with disease activity and severity [154, 158]. However, HASHEMZADEH et al. found while serum BAFF levels were notably elevated in sarcoidosis patients in comparison to the control group, no substantial variations was observed between patients with acute and chronic sarcoidosis [159]. Further research is required to explore the potential of BAFF as a prognostic biomarker. Dysfunction of B-cells and population imbalances in the chronic phase of sarcoidosis may be attributed to impairments in the B-cell signaling pathway [160].

Although anti-B cell therapies have been utilized as fourth-line treatments for sarcoidosis, further exploration and validation of the underlying mechanisms are necessary. This is crucial for advancing B cell-targeted therapies and pinpointing pertinent biomarkers.

#### Intracellular signaling pathways

mTOR pathway plays an important role in pathogenesis of sarcoidosis. Activation of the mTORC1 signaling pathway occurs via signaling molecules such as IFN-γ and IL-17A, along with additional factors secreted by upstream Th1 and Th17 cells, facilitated through the JAK/STAT signaling pathway [11]. Genome-wide sequencing of familial aggregation sarcoidosis shows that the mTORC1 pathway regulates macrophage aggregation, inflammatory pathways, and glycolytic metabolism. After specific knockout of Tsc2 gene in mouse macrophages, chronic activation of mTORC1 pathway causes macrophages to differentiate into M2 type and induce granuloma formation [161]. The mTORC1 pathway can also regulate the metabolic activity of macrophages through cyclin-dependent kinase 4 (CDK4) protein, enhance glycolysis, inhibit NF-κB pathway and apoptosis. The mTOR signaling pathway is pivotal in integrating various environmental cues with inflammatory immune responses within innate immune cells, particularly monocytes, macrophages, and dendritic cells [162, 163]. Furthermore, the mTORC1 pathway may influence autophagy to eliminate abnormal immune cells or harmful components, thereby protecting the body from external threats (including infections) and internal inflammatory damage [164].

After blocking mTORC1 pathway by rapamycin, both granuloma formation and macrophage glycolysis activity were inhibited [165]. A single-centre trial and several case reports have demonstrated the promising effectiveness and safety of mTOR inhibition as a therapeutic approach for sarcoidosis. [166, 167]. However, Pizzini et al. found no correlation between the levels of mTOR activation and the severity of the disease or the requirements for therapy [168].

Therefore, mTOR inhibitors such as sirolimus have emerged as a fourth-line treatment option for tuberous sclerosis. However, the precise mechanisms involved—including the regulatory roles of upstream and downstream molecules—as well as the effects of other mTOR inhibitors like rapamycin or everolimus remain to be elucidated through further research.

Recent studies have shown that an overabundance of inflammatory cytokines, notably IFN-γ, and the ensuing activation of the JAK-STAT pathway, could constitute a potential molecular mechanism driving the pathophysiology of sarcoidosis. Elevated mRNA levels of both IL-6 and IFN-γ have been observed within sarcoid granulomas while IL-6 mainly activating TH17 transcription factor STAT3 and IFN-γ, a cytokine associated with TH1 responses, activating STAT1 induced by STAT4 [169]. In granulomatous inflammation, CD4 + T cells constitute the predominant lymphocyte population associated with this inflammatory response. These cells secrete elevated levels of IFN-γ, IL-2, IL-17, and monocyte-recruiting chemokines. In turn, macrophages secrete a wide range of cytokines including IL-6, IL-12, IL-18, IL-23, and TNF-α, along with T cell chemokines [170]. This mutually reinforcing network of cytokines likely establishes a self-sustaining loop that perpetuates granulomatous inflammation in the presence of pathogenic antigens. While numerous cytokine receptors can activate downstream signaling pathways, some cytokines depend on the JAK-STAT pathway for signal transduction because of lacking intrinsic kinase activity [171].

The JAK protein family encompasses four distinct members: JAK1, JAK2, JAK3, and TYK2. In contrast, the STAT protein family consists of seven different members, namely STAT1, STAT2, STAT3, STAT4, STAT6, as well as STAT5A and STAT5B. IFN-γ can transduce signals via JAK1 and JAK2 to activate STAT1. Meanwhile, IL-6 can transduce signals via JAK1, JAK2, and TYK2 to activate STAT3 [172, 173]. Thus, JAK inhibitors differentially regulate individual cytokines. The potential clinical significance of JAK inhibitors in the treatment of sarcoidosis is indicated by this finding [170]. Reports have indicated that the administration of JAK-STAT inhibitors, notably tofacitinib and ruxolitinib, has resulted in favorable clinical outcomes for the treatment of sarcoidosis, as evidenced by multiple case reports, open-label clinical trials, and observational studies [174].

Rac1 (Ras-related C3 botulinum toxin substrate 1) has also been linked to the pathogenesis of sarcoidosis [175]. Rac1 is activated by upstream macrophages and dendritic cells through the expression of NOD2, TLR4, and TLR9. It identifies and attaches to EPHA2 (ephrin type-A receptor 2) and KALRN (kalirin), thereby engaging in the modulation of various facets pertinent to granuloma formation. Recent investigations have uncovered Rac1’s participation in the assembly of a GTPase complex, which governs the transcription of IL-17A. This regulatory process takes place via the complex’s binding to the IL-17A promoter, mediated by its interaction with the nuclear receptor called RORγt—a pivotal player in the differentiation of pro-inflammatory Th17 cells. Notably, Silencing the Rac gene notably affects both the inflammatory activity of Th17 cells and the production of IL-17A [176]. Conversely, the stimulation of Rac1 via EPHA2 and KALRN takes place subsequent to the activation of diverse pattern recognition receptors (PRRs), such as NOD2 and Toll-like receptors TLR4 and TLR9. The above-mentioned receptors are vital in innate immunity, as they detect invading pathogens and enhance autophagy [11]. Interestingly, Rac1 interacts directly with mTOR and modulates its activity by facilitating the localization of mTORC1 and mTORC2 at specific membranes. This suggests a synergistic role for mTORC1 and Rac1 in granuloma formation [177]. The function of the rac1 pathway in sarcoidosis pathogenesis requires further research and experimental validation.

Besides mTOR, JAK/STAT and Rac1 pathway, NLRP3 pathway is another important pathway activated in granuloma formation of sarcoidosis. It is shown that significant upregulation of the NLRP3 pathway in sarcoid patients in both bronchoalveolar lavage (BAL) cells and miR-223 knockout and NLRP3 knockout mice [178]. The NLRP3 inflammasome pathway’s activation is associated with a worse clinical outcome of the disease [179]. This provides new therapeutic targets and prognostic biomarkers to investigate.

The molecular mechanisms and genetic predisposition discussed above provide several potential biomarkers that may be useful for sarcoidosis. Key biomarkers for sarcoidosis are summarized in Table 2.

**Table 2:** Biomarkers for sarcoidosis

Biomarker	Concise Definition	Origin	Function	Reference
Serum angiotensin-converting enzyme (sACE)	An acid glycoprotein which converts angiotensin I into angiotensin II	Lung tissue	Diagnosis and prognosis	[180]
Serum Amyloid-A (SAA)	Pathogenetic Role in Granulomatous Inflammation	CD68 + macrophages and giant cells in granulomas	Disease activity	[180]
Krebs von den Lungen-6 (KL-6)	Mucin-1 glycoprotein	Type II pneumocytes	Diagnosis and prognosis	[181]
Chitotriosidase (CTO)	An enzyme of the chitinase family	Pulmonary neutrophils and macrophages	Diagnosis, disease activity and progression	[182]
CC-Chemokine ligand 18 (CCL-18)	A CC chemokine produced primarily by antigen-presenting cells	Antigen-presenting cells such as macrophages, dendritic cells, and peripheral blood monocytes	Disease activity and progression	[183]
Carbohydrate antigen (CA) 125	Transmembrane glycoprotein of high molecular weight	Inflamed tissue	Disease activity and progression	[184]
Galectins (Gal)	Proteins binding to β-galactosides induce apoptosis in activated leukocytes	Monocytes, macrophages, endothelial and epithelial cells	Diagnosis and prognosis	[185]
Plasma Matrix Metalloproteinass -7 (MMP-7)	Zinc-dependent endoprotease	Lung epithelium and macrophages	Diagnosis and progression	[183]
Soluble Interleukin 2 Receptor (sIL-2R)	Circulating form of the interleukin 2 receptor	Activated Th1 cells and macrophages	Diagnosis, prognosis and disease activity	[186]
B-Cell Activating Factor B-cell activating factor (BAFF)	A cytokine of the TNF family with a critical role in B-cell development and function	Neutrophils, monocytes, macrophages, and dendritic cells	Diagnosis and prognosis	[159]
Lysozyme	An enzyme with bacteriolytic properties catalyzes the hydrolysis of glycosidic bonds, leading to the degradation of peptidoglycans found in the bacterial cell wall	Monocyte-macrophage complex and epithelioid-like cells	Prognosis	[181]
Neopterin	A non-specific indicator of inflammation produced upon stimulation primarily by IFN-γ	Activated monocytes, macrophages, dendritic cells, and endothelial cells	Disease activity and progression	[187]

## Treatment

The principle of treatment of sarcoidosis is to inhibit granulomatous inflammation, thereby delay the progression of disease to fibrosis and destroy organ function [12]. More than half of patients of sarcoidosis will be spontaneously in remission or asymptomatic all the time, while the other half will suffer a chronic course of disease. Currently, the treatment of sarcoidosis aims to achieve two primary goals: firstly, decreasing the morbidity and mortality risks linked to this condition; and secondly, improving the quality of life, especially with respect to alleviating symptom severity and slowing the progression of physical function decline attributed to the disease [188]. Prompt treatment is necessary for patients with obvious symptom or tendency of organ dysfunction [189].

At present, the medications used for the treatment of sarcoidosis have not undergone rigorous investigation through large-scale randomized controlled trials (RCTs). A majority of these treatments are administered based on "off-label" indications, which are derived from experiences with other diseases. In fact, the management of sarcoidosis is primarily informed by trial results that often exhibit inherent design flaws, particularly in rare cases [190]. The details of the current stepwise treatment options for sarcoidosis are summarized in Table 3. These deficiencies can encompass problems pertaining to sample size, the choice of active disease states, and endpoints that are clinically significant. Furthermore, large studies tend to focus predominantly on pulmonary sarcoidosis, while those involving other organ systems are frequently limited to small-sample studies or case reports [191].

**Table 3:** Therapies for sarcoidosis

	Therapy	Mechanism	Dose	Adverse effects
First-Line Agents	Oral corticosteroids (Prednisone)	Anti-inflammatory	20–40mg/day for 2–6 weeks, tapered to 7.5–15 mg/day over 6–18 month	Diabetes, peptic ulcer glaucoma, cataract, Cushing’s syndrome, and infections
Second-Line Agents	Methotrexate	A folate antagonist which can inhibit purine and pyrimidine metabolism and the synthesis of amino acids and polyamines	7.5–25mg/week	Bone marrow suppression, hepatotoxicity, gastrointestinal reaction, pneumonitis, and infections
	Azathioprine	A purine metabolic inhibitor which can decrease the number of circulating T cells and B cells, and promote the apoptosis of circulating lymphocytes	50–200 mg/day	Bone marrow suppression, hepatotoxicity, gastrointestinal reaction, pneumonitis, and infections
Third-Line Agents	Infliximab	Inhibitors of tumor necrosis factor α, which can eliminate the biological function of TNFα	3–5mg/kg intravenously at weeks 0, 2, then every 4–8 weeks	Activation of tuberculosis, viral infections, allergic reactions possibly with injections, demyelination syndrome, malignancy, and sarcoid-like reactions
	Adalimumab	Inhibitors of tumor necrosis factor α, which can eliminate the biological function of TNFα	40mg subcutaneously every 1–2 weeks	Infections including activation of tuberculosis, malignancy, leukopenia and sarcoid-like reactions
Other Agents	Rituximab	A monoclonal antibody against CD20 + B cells	1000mg intravenously at weeks 0, 2, then every 4–8 weeks	Infections, neutropenia, leukopenia, angioedema
	Sirolimus	A specific inhibitor of the mammalian target of rapamycin (mTOR)	2mg/day	Infections, risk of tumor, thrombopenia, anemia, leukopenia, anaphylaxis
	Tofacitinib	A small-molecule Janus kinase(JAK) inhibitor	5 mg twice/day	Infections and thromboembolic events

### Corticosteroids

At present, oral corticosteroids are still the first-line treatment drugs, which can significantly inhibit the cytokines such as IFN-γ and TNF-α in the pathway of granuloma formation of sarcoidosis. In three placebo-controlled trials with 340 patients suffering from pulmonary sarcoidosis and receiving oral glucocorticoid treatment, a greater proportion showed clinical improvement during short-term follow-up (3–6 months). In addition, a discernible trend was observed indicating a reduction in the number of patients who experienced clinical deterioration over a brief period. Glucocorticoid treatment was favored, as evidenced by a reduced incidence of notable radiographic worsening. Additionally, there was a notable enhancement in pulmonary function among those with initial lung involvement. [192–194]. Seven retrospective cohort studies on cutaneous sarcoidosis indicate that systemic glucocorticoid therapy improves or induces approximately two-thirds of the patient population. [195, 196]. The retrospective studies on cardiac nodular disease and neurosarcoidosis have also shown favorable clinical efficacy in response to glucocorticoid treatment. However, there is a concern regarding the recurrence of symptoms following discontinuation of treatment [197, 198].

The initial dose of prednisone is generally recommended to be 20 to 40 mg everyday [199]. Early studies have shown that a modest reduction of the initial induction dose to 15 mg daily may also improve lung involvement [200]. Later studies showed that patients with poor baseline lung function and higher cumulative doses of hormones had no statistically significant differences in need for second/third-line therapy or number of exacerbations, which suggesting low-dose corticosteroids is also of high clinical value [201]. Doses above 40 mg/day for a long term are not recommended because of high-risk toxicity induced by corticosteroid and tinily clinical benefit [202].

However, prolonged use of glucocorticoids may result in a significant incidence of morbidity. The application of glucocorticoids in treating sarcoidosis is associated with a significant and often underappreciated side effect profile. This includes metabolic complications and an increased risk of cardiovascular disease, which likely escalates with prolonged duration of use [203].

### Corticosteroid-sparing medications

As second-line therapy for sarcoidosis, methotrexate, azathioprine are common alternatives to corticosteroids, which achieve immunosuppressive effects by down-regulating the metabolic activity of CD4 + T cells [199]. Methotrexate is a folate antagonist inhibiting not only purine and pyrimidine metabolism, but also the synthesis of amino acids and polyamines [204]. Doses of 7.5 mg to 15 mg per week are effective in most cases, with higher overall response rates if combined with corticosteroids and lower if used alone [205]. In both a randomized, double-blind, placebo-controlled trial and multiple open-label, prospective, and retrospective studies, methotrexate has shown to exert steroid-sparing effects and is correlated with enhanced lung function [206]. However, a large proportion of sarcoidosis patients do not respond to methotrexate or have toxic reactions, which may reflect certain genetic predispositions to drug efficacy or toxicity [205]. MTX therapy exhibited a minimal occurrence of hepatic or hematological adverse events, with merely 20% of the patient population experiencing worsening of their condition after a year of treatment. [207].

Leflunomide is a dihydroorotase inhibitor that inhibits lymphocyte division, which served as an alternative to methotrexate in treatment of sarcoidosis [208]. Azathioprine, an inhibitor of purine metabolism, exhibits comparable effectiveness to methotrexate in enhancing forced vital capacity (FVC) and diffusion capacity for carbon monoxide (DLCO). It works by reducing the count of circulating T and B cells and facilitating the apoptosis of lymphocytes in the circulation [209]. A randomized study of extended chloroquine treatment indicates that in cases of chronic pulmonary sarcoidosis, high-dose chloroquine improves disease activity. Continued low-dose chloroquine beyond 6 months significantly slows the decline in pulmonary function [210]. However, Hydroxychloroquine has the potential to induce QT prolongation, which may lead to life-threatening arrhythmias. A recently conducted multicenter, retrospective investigation did not uncover a substantial influence of hydroxychloroquine on the corrected QT interval (QTc) in patients with sarcoidosis. [211].

However, the onset time of cytotoxic drugs is longer, and the combination of glucocorticoids is still necessary for patients with acute onset.

### Targeted biologics

Targeted biologics can be used as third-line therapy, mainly including TNF-α antagonists: infliximab (IFX) and Adalimumab. TNF-α, as a product of macrophage activation, is a major cytokine in active sarcoidosis, especially at the site of granulomatous formation [212]. Biologics inhibiting TNF-α have shown promising efficacy in some sarcoid patients. IFX is more effective for extrapulmonary sarcoidosis (including skin, nerve, cardiac sarcoidosis, etc.) and can also be used in all refractory patients [213, 214]. Although large-scale randomized controlled trials (RCTs) are lacking, several retrospective studies and small-sample randomized trials indicate that retrospective studies of infliximab for sarcoidosis demonstrated high success rates in treating central nervous system, skin, lung, and upper respiratory diseases. The most prevalent adverse reactions were non-serious infections such as respiratory infections and shingles [10, 215, 216]. At present, Infliximab is commonly used with a recommended dose of 3-5 mg/kg and maintenance therapy every 4–8 weeks [217]. Recently, several studies indicate that switching to Infliximab biosimilar is safe and effective, providing a cost-effective treatment option for sarcoidosis [218].

In a randomized controlled trial (RCT), adalimumab is effective and safe in the treatment for cutaneous sarcoidosis [219]. It is also reported that adalimumab can improve the symptoms of refractory pulmonary sarcoidosis and ocular sarcoidosis in several studies [220, 221]. In general, adalimumab can not only gradually reduce the dosage of corticosteroids but also be a subset of patients who are resistant to infliximab. Adalimumab has similar side effects to infliximab, primarily infections. Additionally, half of the patients relapsed after discontinuing anti-TNF therapy [222].

Ustekinumab, as monoclonal antibodies specifically inhibiting IL-12/IL-23, while golimumab inhibit TNF-α. There were no significant improvements in lung function in chronic pulmonary sarcoidosis and/or cutaneous, however a nonsignificant improvement of cutaneous sarcoidosis was observed following golimumab treatment [223]. In addition, a case reported Ustekinumab-induced sarcoidosis in a psoriasis patient. This may occur because blocking certain immune pathways can lead to cytokine imbalance in predisposed individuals, resulting in an inflammatory process [224].

### Anti-B cell therapy

Rituximab, as a renowned monoclonal antibody targeting CD20 + B cells, has been investigated in several studies. However, phase I/II trials of rituximab involving 15 patients with refractory pulmonary sarcoidosis, along with several case reports, indicate that rituximab exhibits mixed efficacy [225, 226]. And some clinical cases even reported cutaneous sarcoidosis following rituximab in the treatment of other diseases [155]. Further research is necessary, focusing on the optimization of rituximab dosing and the exploration of the molecular pathways involving B cells in sarcoidosis to clarify its therapeutic trends.

### JAK Inhibitors

Previous experimental studies have shown that tofacitinib, a JAK inhibitor, possesses the capability to inhibit JAK-STAT-dependent signaling pathways and reduce the levels of proinflammatory cytokines, which are known to be involved in the formation of granulomas. Several retrospective studies have demonstrated that oral tofacitinib not only improves cutaneous sarcoidosis but also benefits patients with refractory multiorgan involvement associated with sarcoidosis [227, 228]. An open-label, prospective study exploring tofacitinib as a corticosteroid-sparing agent in pulmonary sarcoidosis revealed that three out of five patients met the primary endpoint, enabling all three to successfully discontinue corticosteroid use without experiencing disease recurrence [229]. Common side effects of tofacitinib include a higher risk of infections and thromboembolic events [230]. Further investigation into the safety and effectiveness of tofacitinib in treating sarcoidosis is necessary through randomized, multicenter studies that utilize placebo controls. In targeted JAK inhibition, isoform-specific inhibitors can enhance therapeutic efficacy and reduce off-target effects. This advancement offers new hope for treating sarcoidosis with JAK inhibitors [231].

### Other agents

With the further elucidation of the molecular mechanism, other therapies targeting various mechanisms of pathology are also under further study. Nicotine decreases cytokine production by acting on NFκB in macrophages and decreases the ratio of Th17/Treg by affecting CD4 + lymphocytes, which can be a possible treatment for sarcoidosis [232]. The mTORC1 pathway is thought to contribute to abnormal autophagy in sarcoidosis. It has been reported that after continuous administration of sirolimus (2 mg/d) for 10 months in a patient with refractory pulmonary sarcoidosis, the cough symptoms were significantly relieved, and the lung CT images showed significant regression of the lesions. Several clinical cases suggest that mTOR inhibitors may have the potential to treat sarcoidosis [233]. Abatacept is a fusion protein of CTLA-4 and immunoglobulin, which can down-regulate T cells co-stimulatory signal and inhibit activation of T cells. So, interfering with CTLA-4 using abatacept might be a new option of therapy in sarcoidosis, which is in clinical trials currently [234].

## Summary

The molecular mechanism of granuloma formation is complex involving various immune cells. Macrophages (monocytes) are activated upon antigen stimulation and secrete various cytokines, such as TNF-α, IL-18, IL-23, and IL-2. Th0 cells undergo differentiation to Th1 cells under the influence of TNF-α and IL-18 or to Th17 cells under the influence of IL-6 and TGF-β. Activated Th cells produce a large amount of IFN-γ and may partly and persistently activate macrophages through the JAK-STAT signaling pathway. Blocking the mTORC1 pathway can inhibit macrophage differentiation into the M2 type and prevent granuloma formation. Additionally, activated Th cells continue to secrete various chemokines to recruit immune cells. Furthermore, Th cells proliferate and fuse to form epithelioid cells or multinuclear giant cells and consequently promote antigen phagocytosis. Sarcoidosis can be spontaneously alleviated upon antigen clearance. Macrophages may continue to exhibit phagocytosis and induce chronic inflammation if the antigens are not effectively cleared. Inhibitory regulatory mechanisms, including reduced functionality of Treg and Th2 cells, are crucial in the chronic inflammatory processes related to sarcoidosis.

The balance among Th1, Th17, Th17.1, and Treg subsets has become a focal point of research regarding the formation and maintenance of granulomas in sarcoidosis. Their balance especially Th17/Treg rebalance is essential in sarcoidosis and represents a promising direction for future research on biomarkers and therapeutic agents. Certain cohort studies have revealed a correlation between diminished Treg functionality and the disruption of the balance between Th17 and Treg cells, which is associated with the degree of sarcoidosis severity and its prognostic implications. However, the research is limited to fundamental studies on animals at present, and further clinical intervention studies are needed to determine whether manipulating Treg function and Th17/Treg cell rebalance can improve clinical outcomes. Additionally, autophagy and its regulatory molecules, such as mTORC1, may offer a new treatment approach for the treatment of sarcoidosis.

The main focus of sarcoidosis treatment is inhibition of granulomatous inflammation. Corticosteroids and immunosuppressor are first-line and second-line therapy for sarcoidosis. Targeted biologics such as Infliximab and Adalimumab can be used to target TNF-α for treatment of sarcoidosis when corticosteroids and immunosuppressor are ineffective. In studies investigating the efficacy of TNF-α inhibitors (Infliximab and Adalimumab) in treating sarcoidosis, some cases have shown suboptimal responses. This may be related to polymorphisms in the gene responsible for transcribing TNF-α. The genotyping of the TNF-α G-308A polymorphism may serve as a possible indicator for predicting the therapeutic efficacy of TNF-α treatment in sarcoidosis [235]. In recent years, research has demonstrated that the pan-JAK inhibitor tofacitinib exhibits favorable safety and efficacy in sarcoidosis, particularly in cases of cutaneous sarcoidosis. However, the potential effectiveness of new selective JAK1 inhibitors, such as upadacitinib, in treating sarcoidosis may represent a promising direction for future studies.

In summary, advancements in the immunopathogenesis and molecular pathways of sarcoidosis provide new promising agents and biomarkers. However, numerous challenges remain on the horizon. It is crucial to consider multiple factors when developing new therapeutic strategies for sarcoidosis, aiming to improve care while reducing the risk of adverse reactions.

## Data Availability

Not applicable.
